# The Via Media of Dietetics

**Published:** 1909-06-05

**Authors:** 


### The Hospital

A Journal of

The Medical Sciences and Hospital Administration.

New Series. No. 118, Vol. V. [No. 1190, Vol. XLVI.] SATURDAY, JUNE 5, 1909.

### THE VIA MEDIA OF DIETETICS.

It is a maxim that the curability of any given disorder stands in an inverse ratio to the number of its "cures." This is natural. No one, having witnessed the effect of quinine upon malaria, is particularly concerned to continue experiments with other drugs. Conversely inveterate diseases have a large following of "cures," for human hopefulness is, fortunately, as inveterate as the least amenable of maladies, and stimulates humanity to the perpetual exploiting of possible remedies, often enough against the dictates of reason. These observations apply both to sufferers from the organic maladies whose consideration fills the pages of text-books on medicine, and to the immense class of persons who labour not so much under disease as dis-ease. These last are not technically sick in so far as their bodily functions are tolerably well discharged, though not without unaccountable vagaries from day to day—vagaries in themselves insignificant, yet in sum substantial enough to discount enjoyment of life while not jeopardising physical existence. Their complaint is rather of unexplained dis-ease, lack of placidity, absence of the *joie de vivre* which is the high prerogative of healthy youth, and is seen at its best in the explosive hilarity of a six-months' puppy.

This is a venerable disability. How venerable may be judged from the immense literature of the philosophies which deal with it and endeavour to point the way to those who are lost in its mazes. Here is Epicurus on the subject: "There are two good things of which our highest good or chiefest felicity doth consist, viz. to have the mind free from perturbation and the body free from pain; and so that these goods be full and above the capacity of increase. For how can that which is full be increased? If the body be immune from all pain, what addition can be made to that indolency? If the mind be constantly serene and imperturbed, what addition can be made to that tranquillity? Now, seeing that this tranquillity of mind and indolency of body do constitute the chief felicity of man, nothing can more concern us than to consider those things which conduce to the attainment and conservation thereof: insomuch as while we have that we have all things; and while we want it, all we do is to attain it, though, for the causes aforesaid, we seldom do attain it." Certainly in this particular humanity has not changed much in the last two thousand years. There are still thousands of men and women beneath the sun searching for this "indolency" (in the archaic sense) of body, and tranquillity of mind so highly rated by Epicurus.

The paths along which such victims seek to achieve their liberty are, like the manifestations of their dis-ease, legion. Having traversed the pharmacopœia in vain, and Harley Street without advantage, they are ripe—and small blame to them—for the missionaries of any cult which still enables them to nurse hope of relief; and it is a matter partly of chance, partly of temperament, whether in the next step of their evolution they enter the ranks of theosophy, food-faddism, Christian Science, homœopathy, or some other competing creed. We are concerned for the moment with food-fads, our text being some lectures upon Parcimony in Nutrition, delivered by Sir James Crichton-Browne last year, and now issued in book form.*

It may, we think, be taken for granted that so long as bodily and mental health remain unimpaired—that is, so long as a man continues to be unconscious of the machinery of his daily life—he cares little what he eats and drinks, but follows the invitations of his appetite, in so far as they consort with his pocket, ungrudgingly and with a light heart. Therefore the fact that a man begins to be curious about his diet is good evidence that all is not well with his mechanism (excluding, perhaps the small class of people who diet themselves from fears of obesity and for their figures' sake alone). We are met, then, with an army of people suffering in a fashion little or not at all accounted of in works on medicine, folks who are constantly on the look-out for some explanation of the dis-ease within them, and who in their search after knowledge learn early that there are such things as malignant metabolic poisons. For does not every penny print assure them in ominous terms that this, that, and the other symptom is one of the metabolic poisons whose head is Uric Acid? Thus primed they probe further and derive, from an ample literature, a knowledge of

* "Parcimony in Nutrition," by Sir James Crichton-Browne. Funk and Wagnalls, London and New York. 1909. Pp. 111. 3s.

the virtues of vegetarianism, Fletcherism, and the like. Forthwith they undertake the enterprise, and, commonly, with some temporary success. The detached observer may ask himself the reason of this advantage, which, though transient as a rule, must, we think, be admitted an existence. The probable explanation is that the change of diet acts for a while after the fashion of that vague but important therapeutic item, an alterative, the body after becoming "stale" with monotony being benefited by the change of diet. However, it be, there is no doubt that the passing advantage often attending the assumption of a dietetic fad has produced a school of dieticians whose motto is parsimony in nutrition, a school confirmed in their beliefs by the researches of Chittenden upon the blessings of a diet falling far short of what custom and inclination have prescribed. Sir James Crichton-Browne, in the lectures to which we refer, enters a caution against the too ready acceptance of these experimental studies as a guide for practical living. His book repays perusal, for it is balanced and sane. On the whole, he is of Bacon's opinion: "Celsus could never have spoken it as a physician had he not been a wise man withal, when he giveth it for one of the great precepts of health and lasting, that a man do vary and interchange contraries, but with an inclination to the more benign extreme: use fasting and full eating, but rather full eating; watching and sleep, but rather sleep; sitting and exercise, but rather exercise, and the like. So shall Nature be cherished and yet taught masteries."

